# Are Janus Kinase Inhibitors Superior over Classic Biologic Agents in RA Patients?

**DOI:** 10.1155/2018/7492904

**Published:** 2018-05-10

**Authors:** Przemyslaw J. Kotyla

**Affiliations:** Department of Internal Medicine and Rheumatology, Medical Faculty in Katowice, Medical University of Silesia, Katowice, Poland

## Abstract

The Janus Kinases (JAKs) are a family of intracellular tyrosine kinases that provide transmission signals from cytokine, interferons, and many hormones receptors to the nucleus resulting in synthesis of many biologically active compounds and changing cell metabolism and function. That was theoretical background to synthetize the JAK inhibitors (Jakinibs). In recent years a substantial battery of evidence has been collected indicating the potential role of Jakinibs to interact with the specific elements of the immune system, therefore changing the inflammatory response. JAK kinase blockade offers a unique opportunity to block most of the key cytokines enabling the deep interaction into immune system functioning. Following discovery first Jakinibs were intensively studied in various forms of autoimmune diseases, including rheumatoid arthritis, and finally two Jakinibs tofacitinib and Baricitinib have been approved for the treatment of rheumatoid arthritis. Some clinical data indicated that under special circumstances Jakinibs may be even superior to biologics in the treatment of RA; however this suggestion should be verified in large clinical and observational studies.

## 1. Introduction

Rheumatoid arthritis (RA) is a chronic, devastating polyarthropathy with symmetrical involvement of peripheral joints [1]. Synovial inflammation in joints directly leads to cartilage damage with formation of bone erosions followed by joint space narrowing. The disease leads to disability particularly if poorly controlled and is also a leading cause of premature death. Having a prevalence of 1% RA is recognized as the most common form of inflammatory polyarthropathy. The disease affects three times more females than men. The etiology of the disease although not fully understood comprises a variety of factors including environmental, genetic, and lifestyle related factors [2]. Recent advances in genetic studies using single nucleotide polymorphisms enabled the characterization of more than a hundred loci associated with rheumatoid arthritis risk. Most of them are directly involved in proper immune system functioning; some of them already played a role in pathogenesis of the other immune driven disorders [3]. At the current level of knowledge the HLA system (particularly HLA-DRB1) is believed to be one of the most important players, strongly supporting hypothesis of antigens or (and self-antigens) recognition in RA pathogenesis. This region encodes many important molecules and transmitters which are directly involved in areas such as immune processes as costimulation, T cell recognition of antigens, cytokine receptors expressions, posttranslational citrullination, and synthesis of intracellular regulatory molecules directly responsible for immune signals transmitting [4].

The inflammatory states start with breaking the tolerance of T and B cells against self-antigen (antigens). This ultimately leads to uncontrolled immune response [5]. Recent advances in understanding the pathogenesis highlighted the role of the cytokine network in the initiation and progression of the disease [6–8]. This led to development of a novel class of drugs for rheumatoid arthritis directly targeting cytokines and costimulatory molecules or causing depletion of whole lines of immune cells [9]. This new class of drugs called biologics or biological DMARDs (bDMARDs) revolutionized treatment of RA [10–12]. This kind of treatment has, however, some limitations. The most important one is primary or secondary lack of efficacy. It is estimated that up to 30% of patients still do not respond adequately to the treatment, which requires switching the treatment to the second-line agents [13]. The other important issue is biologics-related toxicity, increased risk for severe infection, and infusion-related adverse effects [14]. With the exception of abatacept and rituximab, all agents available so far interact with cytokine network (anti-TNF, anti-IL-6) [15]. All of those agents are high molecular weight proteins with complicated molecular structure and they have to be administered parenterally. The other important consequence that should be kept in mind is the fact that biologics may generate immune system response that leads to the formation of neutralizing antibodies, causing secondary lack of efficacy [16, 17]. Given the efficacy of biologics against different targets, the open question remains whether patients who do not respond to first-line biologic (usually anti-TNF) may differentially respond to another drug from the same group (another TNFi) and why some patients respond to anti-TNF although they do not respond to anti-IL-6 and vice versa? This clinical observation gives some insight into pathogenesis of RA indicating diversity of causative factors, cytokines, and transmission molecules creating a unique immunological environmental in a given patient.

This limitation may be overcome by the targeted synthetic DMARDs (tsDMARDs) or biologics that should be considered when treatment target is not achieved with conventional synthetic DMARD and poor prognostic factors are present. Current recommendations, however, indicate to start treatment with bDMARDs [18]

Due to their crucial roles as signal transducers downstream of cytokine receptor activation, the Janus Kinase (JAK) family of tyrosine kinases have attracted much attention since their discovery more than 20 years ago [19–21]. Cytokine receptors are specific type of receptors since they lack intrinsic protein kinase domains and entirely rely on the enzymatic activities of Janus Kinase attached to cytoplasmatic part of cytokine receptors [22]. This makes receptor-JAK interaction the most important step in signal transmission. In line with it JAK inhibition blocks action of all dependent cytokines* (“many birds with one shot”).*

## 2. The Janus Kinases Structure and Function

The Janus Kinases (JAKs) are a family of intracellular tyrosine kinases that provide transmission signals from cytokine, interferons, and many hormones receptors to the nucleus resulting in synthesis of many biologically active compounds and changing cell metabolism and function [23]. With this ability to transmit cytokine-related signals JAKs play a key role in proper function of innate and adaptive immune systems as well as an important role in such pathophysiological processes as hematopoiesis, immune cells development, and many others [24]. In mammalians the JAK family consists of four members (JAK1, JAK2, JAK3, and TYK2) that are specifically associated with different types of cytokine receptors [25]. Cytokine (ligand) binding activates JAKs which in turn facilitate binding of the other transmission molecules, namely, STAT (signal transducer and activator of transcription). STATs are DNA-binding proteins which underwent phosphorylation, which allows dimerizing them, translocating to the nucleus, and regulating gene expression. Ligand-JAK activation is not restricted to one cytokine, but one specific JAK could be activated by several cytokines. JAK1, JAK2, and TYK2 are expressed by many cells while JAK3 expression is restricted to hematopoietic, myeloid, and lymphoid cells [26]. In line with this JAKs play a crucial role in normal hematopoiesis and kinases malfunction results in hematopoiesis dysfunction and immunodeficiency [27].

**Figure 1:**
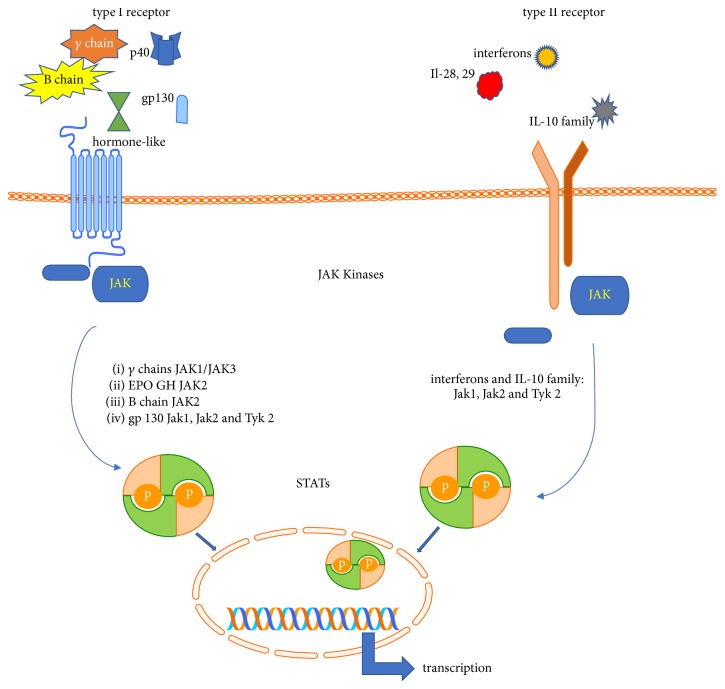
JAK/STAT transmission pathways from type I and type II cytokines receptors. Type I: several cytokines utilize type I cytokine receptor: (i) Il-2, Il-4, Il-7, IL-9, Il-15, and Il-21 that use gamma chain of cytokine receptor; (ii) GM-CSF Il-3 and Il-5 utilize beta chain of cytokine receptor; (iii) IL-6, Il-11, and Il-27 are cytokines that interact with receptors containing gp 130 subunit; (iv) Il-12 and Il-23 with common p40 subunit; (v) erythropoietin, thrombospondin G-CSF GH, and leptin also use type I receptor homolog. TYPE II: (i) type II receptors are main receptors for interferons *α*, *β*, and *γ*; (ii) IL-28 and IL-29 (IFN lambda) also interact with type II; (iii) Il-10 family cytokines are ligands for type II.

JAKs are an essential part of receptor activity. The majority of known cytokine receptors utilize JAKs as a catalytic center to transmit signals from cytokines and hormone and growth factors to the nucleus to promote transcription of ligand-related genes. Based on structural homologies in receptor sequences and similarities in structure of cytokines, cytokine receptors are typically divided into two subgroups, namely, class I and class II [28, 29]. Class I and class II receptors are protein complexes expressed on the surface of cells. They consist of one to four receptor chains. The typical structure of receptor consists of an extracellular cytokine R homology domain (CHD) and a sequence responsible for cytokine binding. Based on structural differences within CHD cytokines receptors may belong to a class I or class II family [30]. Class I receptor chains have two disulfide bridges linking cysteines in two chains of receptors. But the most important mark of class I is the presence of a highly conserved WSXWS motif [28]. Contrary to class I, class II receptors may form only one disulfide bridge as receptor chains have only one cysteine pair in their CHD [31]. Based on the presence of signal transducing chains that build the active receptors and are entirely responsible for ligand recognition the class I receptor family may interact with four cytokine subfamilies and one hormone-like cytokine receptors. The common gamma family (*γ*c) transmits signals from IL-2, IL-4, IL-7, IL-9, IL-15, and IL-21 [32]. The common beta family (*β*c) is involved in activating GM-CSF, IL-3, and IL-5 followed by family receptors that utilize gp 130 protein and act as a transducer for IL-6, IL-11, IL-31 (gp130 homolog), IL-35, and IL-27. The last family in this group consists of Il-12 and Il-23 interleukins receptors for heterodimeric cytokines that share the common subunit p40 [33, 34]. Type I receptors are also involved in recognition of signals from some hormone-like cytokines as erythropoietin, thrombopoietin growth hormone, and leptin (Figure 1) [35].

The type II cytokines consist of more than 300 signaling molecules including mainly but not exclusively interferons types I, II, and III but also cytokines of IL-10 family (IL-10, IL-19, IL-20, IL-22, IL-24, and IL-26) [29]. Although many cytokines, hormones, and growth factors interact with receptors coupled with JAKs, each cytokine receptor family interacts with the specific JAK/JAK composition [36]. Hormone-like cytokines interact with receptors that transmit signals through homodimers of JAK2. The *γ*c family utilized heterodimers of JAK1 and JAK3, and the *β*c family uses JAK2. Type II cytokine receptors are linked to JAK1, JAK3, and TYK2 [37, 38]

**Figure 2:**
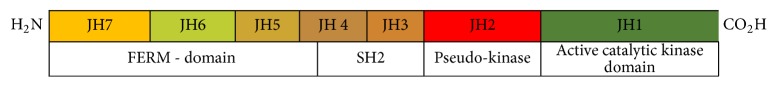
Structure of JAK kinase. Seven JAK homology regions (JHs) built the structure of four structural domains. JAK active catalytic domain is regulated by pseudokinase, which exerts regulatory role over catalytic centre of enzyme. The remaining domains (FERM and SH2, resp.) are responsible for maintaining the structure of kinase and for interaction with cytokine receptor.

JAKs share seven homology domains termed the JAK homology (JH). JH1 and JH2 are located at the C terminal end of the enzyme encoding a kinase and a pseudokinase, respectively [39]. Contrary to JH1, JH2 domain is characterized by dual kinase specificity and acts as a regulator of JH1 kinase domain activity [40]. The other domains do not encode enzymatic domains but are involved in binding the kinases to the cytoplasmic tails of receptors which contain box 1 and box 2 motifs; both of them are required for proper JAK engagement. Structure of box 1 and box 2 varies substantially between receptors; however proper function and structure are essential for receptor-JAK interaction [41]. This binding part of the enzyme consists of four domains, specifically JAK-FERM (JH5-7) and SH2- (Src homology-2-) like domains (JH3-4) (Figure 2) [37]. The structure of JAK-FERM resembles the canonical FERM and consists of three subdomains, ubiquitin-like F1, acyl-CoA-binding protein like F2, and pleckstrin homology domain-like F3. There are however many differences: JAK FERMs are characterized by a longer L1 linker usually placed between 29 and 42 amino residues and the SH2 domain is packed against an F1 *α*1 helix F3-SH2 linker and L3 linker. Some deviations may be observed in regard to F2 domains with additional residues F2*α*1 and F2*α*2. It is speculated that this elongation facilitates binding JAK FERMs to the cytokine receptor [42].

## 3. CytoR-JAK-STAT Signal Transmission Pathway

Receptors associated with JAK kinases are dimers, assembling with two receptor chains. After activation two chains dimerize to mount the active receptor. After ligand ligation by receptor, receptor subunits are oligomerized leading to reorientation of receptor-associated JAK enabling them to take a position that facilitates their transphosphorylation and activation [43]. Phosphorylated JAK then phosphorylates tyrosine residues within the cytoplasmic tail of the receptor leading to conformational changes enabling creation of docking sites for signal transducers and activators of transcription (STAT) proteins acting as downstream regulatory factors [44]. Interaction between STATs and receptor chains is realized by interaction between phosphotyrosine recognition domains on receptor chain and SH2 domains expressed by STAT proteins. Depending on the type of receptor and structure and position of phosphotyrosine domains in the receptor tail one of seven STAT proteins (STAT1, STAT2, STAT3, STAT4, STAT5a, STAT5b, or STAT6) may be recruited by the receptor. Close proximity of JAK to the STAT enables it to activate STAT followed by STAT translocation to the nucleus, where it acts as transcriptor factor [45]. This process is regulated by the Suppressor of Cytokine Signaling (SOCS) family of negative regulators, which modulate signaling by inactivation of the Janus Kinases (JAKs), preventing access of STATs to receptor binding sites, blocking signaling proteins access to the proteasome [46, 47]. This shows the potential of SOCS family members to block multiple cytokine-induced signaling pathways. Obviously the physiological effect depends on which cytokine action is abolished [48].

## 4. Primary Immunodeficiencies and Autoimmunity

Primary immunodeficiencies may serve as a model to understand a key role of JAKs in normal immune response as well as to understand pathophysiological consequences of JAK blockade. Mutations in JAKs lead to development of myeloproliferative diseases but also lead to autoimmunity and immunodeficiency states [49]. As an example we can consider a loss of function mutation in JAK3 [50–52]. Working together with JAK1, JAK3 is responsible for transmission of signals provided by *γ* chain receptors thereby enabling physiological activity of cytokines (IL-2, IL-4, IL-7, IL-9, IL-15, and IL-21)—cytokines that utilize *γ* chain receptors to transmit their signals [53]. JAK3 blockade resembles (and in fact is clinically indistinguishable from) a pure switch off mutation in *γ* chain resulting in severe combined immune deficiency (X-linked SCID) [54]. This disease is characterized by a combination of impaired T cell development and reduced immunoglobulin synthesis leading to the development of a clinical picture with severe diarrhea, recurrent severe infection, and atopic dermatitis being the most important ones [55].

Lack of immunoglobulin synthesis is the result of impaired cooperation between T and B cells, although the structure of B cells is not impaired [56]. This illustrates the basic role of *γ* chain transmission in the proper function of immune system. Specifically, reduced IL-15 signaling results in impaired development of T and NK cells (T^−^NK^−^B^+^ SCID). In contrast, blockade of signaling from the interleukin-7 receptor specifically impairs T cell development, leading to T^−^B^+^NK^+^ SCID, since interleukin-15 signaling, which is required for NK-cell development, is maintained. Finally impaired IL-21 signaling substantially explains the nonfunctional B cells in this disease [57–59]. Contrary to this, elevated levels of the other cytokines, which transmit their signals through JAKs, may be recognized as pathogenic factors or at least serve as a marker of autoimmune diseases. For example in early rheumatoid arthritis synovial fluid is characterized by high amount of IL-4. Recent data suggest the role of IL-9 in such autoimmune disorders as systemic lupus erythematosus, multiple sclerosis, psoriasis, rheumatoid arthritis, atopy, and inflammatory bowel disease [60]. The other striking example of the substantial role of JAK-STAT function is the autosomal dominant hyper IgE (HIES or Job's syndrome) syndrome that is characterized by eczema, recurrent pneumonia, chronic mucocutaneous candidiasis, and very high level of IgE [61, 62]. The syndrome is caused by mutation in the DNA-binding Src homology 2 domain of STAT3 [63]. STAT3 activity is essential for cytokines that orchestrated development of IL-17 dependent immune cells (although signaling by IL-17 is not mediated via JAK-STAT system) [64].

## 5. Neoplasms

The crucial role of the JAK-STAT system in host defense, immune response, and autoimmunity suggested the role that JAK may play in cancerogenesis. It was established that several gains of function mutations (activation mutation) in JAK1, JAK2, and JAK3 are entirely responsible for hematopoietic disorders such as T and B cell acute lymphocytic leukemias, acute myeloid leukemia, polycythemia vera, essential thrombocytopenia, or Hodgkin Lymphoma [65].

This is especially true for JAK2 activation mutation, where the most frequent mutation V617F is seen in over 95% of cases of polycythemia vera and up to 57% in patients with primary myelofibrosis or essential thrombocythemia [66]. Augmented cytokine signaling is also a hallmark of some solid tumors [67]. With the key role of STAT3 that is now commonly accepted to support tumorigenesis by various mechanism of immunocompetent cells cross talk. This indicates the role that the JAK-STAT axis plays in neoplasm development but also indicates possible medical interventions.

## 6. Rationale for JAKs Blockade in the Treatment of Autoimmune Diseases

In recent years a substantial battery of evidence has been collected indicating the potential role of JAK kinase inhibitors (Jakinib) in interacting with the specific elements of the immune system, therefore changing the inflammatory response. JAK kinase blockade offers a unique opportunity to block most of the key cytokines enabling the deep interaction into immune system functioning. There are however many limitations of such a treatment. Firstly the first generation of Jakinibs (pan-inhibitors) target many of the known JAKs. Taking into account the fact that JAKs are a group of signal transmitters, panblockade may not only result in reduction of inflammatory response but also contribute significantly to development of serious adverse events, toxicity, increased risk of infection, bone marrow suppression, and higher rate of cardiovascular events [68]. Secondly JAKs blockade may potentially reduce anti-inflammatory response provided by anti-inflammatory cytokines such as IL-10 (JAK1/TYK2) and IL-4 (JAK1/JAK3). Moreover JAK cannot transmit signals provided by TNF, IL-1, IL-8 TGF*β*, MCSF, and IL-17, which maintain normal immune response to infectious agents on one side but reduce efficacy of drugs in treatment of some autoimmune diseases on the other. This limitation should be borne in mind when starting treatment with Jakinibs.

Tofacitinib was the first Jakinib approved for treatment of autoimmune diseases in humans. The background for its introduction to clinical practice was the role of JAK3 in transmission of many inflammatory stimuli provided by type I and II cytokines. Initially it was believed that tofacitinib is a selective Jakinib blocking only JAK3. Therefore it may exert high therapeutic potential parallel with an acceptable adverse effect profile. Subsequently it was clear that tofacitinib also blocks JAK1 and to a lesser degree JAK2 [69]. This paradoxicality may be an advantage of tofacitinib as mild inhibition of JAK1 and JAK2 does not change the safety profile of the drug but provides enhanced efficacy of the compound in the treatment of autoimmune diseases. In line with this finding FDA approved tofacitinib for patients with RA refractory or intolerant to methotrexate. This was based on a synthesis of data accumulated from phase II and III trials where tofacitinib was extensively tested against methotrexate and placebo in patients with rheumatoid arthritis [70]. Studies in phase II where tofacitinib 5 and 10 mg daily was compared to placebo showed significantly higher ACR 20 response rate at week 12. The improvement in tofacitinib group was seen as early as weeks 1 and 2 and the therapeutic effect was sustained to the end of the treatment. Patients treated with tofacitinib showed higher ACR 50 and ACR 70 response rate versus placebo arm and the effect was seen in both tofacitinib doses [71–73]. Moreover in trials with RA patients tofacitinib was noninferior to a TNF inhibitor—adalimumab [72]. This is an important finding as JAK3 kinase does not directly transmit signals provided by TNF*α* and anti-TNF*α* biologics actually increase level of this cytokine [74]. This also gives an interesting insight into pathogenesis of RA, suggesting that disease is not driven by a single cytokine but it is the result of interaction of several proinflammatory cytokines building together a proinflammatory milieu. Phase III trials confirmed observations driven from phase II [75]. Significant improvement in almost all measured outcomes has been recorded in tofacitinib group regardless of the previous treatment (biologic DMARDs naïve, biologic DMRDs resistant) [76]. Subsequent studies in phases III and IIIb/IV confirmed that tofacitinib treatment is noninferior to standard care with TNFi (adalimumab) [77, 78].

Inhibition of inflammation is not obviously a target but rather the way of the treatment. The real target of treatment is to halt structural damage of joint and prevent disability. In this field tofacitinib also showed high therapeutic potential halting the progression of joint damage [79].

Deep interference in the immune system has to bring many safety issues, as a blockade of JAK-STAT transmission inhibits the action of several cytokines involved in normal immune response thus reducing organism self-defense. Therefore this issue has also been extensively studied. The adverse events (AE) incidence ratio did not differ significantly between tofacitinib and placebo arms. The most common AEs in the initial phase of study (months 0–3) were diarrhea, nasopharyngitis, headache and urinary tract infection within later phase (months 3–6), upper respiratory tract infections, nasopharyngitis, and bronchitis [80]. Of note is a smaller increment in hemoglobin concentration observed in tofacitinib group (10 mg bid) in comparison with smaller dose tofacitinib group (5 mg bid). That may be due to direct blockade of erythropoietin signals in patients on higher doses of drug that superimposes beneficial effect of inflammatory cytokines with blockade on the hematopoietic system.

Blocking of signal transmission by JAK inhibition may be therefore potentially dangerous. The special issue is JAK inhibitors selectivity as JAK1 and JAK2 blockade are lethal in the mouse [81, 82]. Fortunately, contrary to the permanent inhibition of JAKs that would lead to severe immunodeficiency, many accumulated data suggest that temporary and reversible JAKs inhibition may provide safe and efficacious treatment for many autoimmune diseases.

## 7. BARICITINIB: A JAK1/JAK2 Inhibitor

The therapeutic potential of reversible blockade of JAK1 and JAK2 in autoimmune diseases has been intensively studied. JAK1 is associated with *β* chain of IL-2 receptor as well as the other cytokines as interferons, *γ*-chain cytokines, interleukins of IL-10, IL-12 family, and those that utilize gp130 receptor subunit. JAK2 is coupled with receptors expressed on variety of hematopoietic cells and is involved in transmission of signals provided by erythropoietin, thrombopoietin, GM-CSF, IL-3, and IL-5. Therefore JAK2 function is essential for hematopoiesis. Selective blockade of JAK1 and JAK2 may cover many of signaling transmission pathways, most of them involved into pathogenesis of RA. This was a background to develop a second generation of Jakinibs. Baricitinib, an oral selective inhibitor of JAK1 and JAK2, has proven its safety and efficacy in RA patients naïve to csDMARD therapy with no prior bDMARD [83] with inadequate response to methotrexate [84, 85] and conventional DMARD [86] and patients with an inadequate response to or side effects associated with the treatment with one or more tumor necrosis factor inhibitors and/or the other biologic DMARDs [87]. Moreover, as it was shown in phase 3, double-blind, placebo- and active-controlled trial with 1307 patients with active rheumatoid arthritis and treatment with Baricitinib showed superiority over adalimumab as Baricitinib for the ACR20 response and mean change in DAS28-CRP at week 12 [88]. This indicates that Baricitinib may be more potent drug for rheumatoid arthritis than tofacitinib which is characterized by similar potency in ACR responses as compared to adalimumab [78]. The results, however, should be interpreted with caution as the studies differ with regard to their design. This finding is not surprising in light of the potency of Baricitinib to block JAK1, a key transmitter of signals provided by IL-6. As it was shown in Adalimumab-Tocilizumab study, Il-6 blockade may provide more powerful therapeutic effect than inhibition of TNF*α* [89]. Moreover treatment with Baricitinib was associated with halting the progression of bone erosions to a similar extent to that observed during the treatment of TNFi-adalimumab [88]. What is of special interest is that the therapeutic response is maintained through the treatment [90]. Quite recently Fleischmann et al. performed a post hoc analysis of two phase III studies of Baricitinib 4 mg in classic synthetic DMARD-resistant patients with RA, which showed similar response to treatment in young and elderly patients [91].

The therapy with Baricitinib may potentially bring some safety issues. As far as safety of the treatment is concerned, rates of adverse events were more frequent with Baricitinib than with placebo but similar to that observed in the adalimumab group. But what may be of special interest in the light of deep immunosuppression is that rates of serious infection were similar in the placebo, Baricitinib, and adalimumab groups.

## 8. Conclusions

The Janus Kinase family inhibitors represent a novel group of small molecules successfully introduced to the treatment of RA and other autoimmune diseases. With unique potential to inhibit signal transmission provided by a wide branch of inflammatory cytokines these compounds may provide a stable and pronounced therapeutic effect. As the drugs are still under clinical judgement with only two JAK inhibitors (tofacitinib and Baricitinib) currently approved for clinical use, it is too early to speculate whether these compounds may substitute biologics that are currently being used. There are however some advantages over classical biologics that Jakinibs potentially may have. The first one is the blockade of a wide spectrum of cytokines that may cover many existing and potential inflammatory pathways. As it comes from lessons from anti-TNF, inhibition of single cytokine does not guarantee therapeutic effect in all patients with RA. So, blockade of multiple cytokines with one agent may be of special interest. Moreover the therapeutic potential of single biologics has a tendency to exhaust while continuing the treatment leading to secondary lack of efficacy. It is largely due to formation of anti-drug antibodies which are able to neutralize activity of biologics. It may be also speculated that inhibition of one cytokine pathway contributes to activation of alternative inflammatory pathways which do not use the cytokine currently blocked. Secondly Jakinibs are small nonprotein substances lacking potential to generate antidrug response and therapeutic effect may be more stable. It is also worth underlining that treatment with Jakinibs is not associated with allergic reaction, making this treatment safer compared with typical biologics. Similar to biologics (in some study higher) the anti-inflammatory potential of Jakinibs seems similar to biologics (in one study higher) is undoubtedly a great advantage, but again it is too early to draw the final conclusion on the base of the results from one study [78, 88]. Finally Jakinibs as small chemical compounds are easy to synthesize, which indicates that in the future the price for treatment may be substantially lower than biologics with advanced and complicated chemical structure.

At the moment we have been given a new therapeutic option for patients who do not respond to TNF inhibitors, with the hope that our potential to reach targets in RA would be easier.
